# Human Breast Milk Bacteriome in Health and Disease

**DOI:** 10.3390/nu10111643

**Published:** 2018-11-03

**Authors:** Anna Ojo-Okunola, Mark Nicol, Elloise du Toit

**Affiliations:** 1 Division of Medical Microbiology, Department of Pathology, University of Cape Town, Observatory, Cape Town 7925, South Africa; mark.nicol@uct.ac.za (M.N.); elloisedutoit@gmail.com (E.d.T.); 2 Division of Medical Microbiology, National Health Laboratory Service, Observatory, Cape Town 7925, South Africa

**Keywords:** bacteriome, human breast milk, bacterial community, mastitis, human immunodeficiency virus (HIV), cancer

## Abstract

It is well-known that, beyond nutritional components, human breast milk (HBM) contains a wide variety of non-nutritive bio-factors perfectly suited for the growing infant. In the pre-2000 era, HBM was considered sterile and devoid of micro-organisms. Though HBM was not included as part of the human microbiome project launched in 2007, great strides have been made in studying the bacterial diversity of HBM in both a healthy state and diseased state, and in understanding their role in infant health. HBM provides a vast array of beneficial micro-organisms that play a key role in colonizing the infant’s mucosal system, including that of the gut. They also have a role in priming the infant’s immune system and supporting its maturation. In this review, we provide an in-depth and updated insight into the immunomodulatory, metabolic, and anti-infective role of HBM bacteriome (bacterial community) and its effect on infant health. We also provide key information from the literature by exploring the possible origin of microbial communities in HBM, the bacterial diversity in this niche and the determinants influencing the HBM bacteriome. Lastly, we investigate the role of the HBM bacteriome in maternal infectious disease (human immunodeficiency virus (HIV) and mastitis)), and cancer. Key gaps in HBM bacterial research are also identified.

## 1. Introduction

Human breast milk (HBM) is a complex, specific, physiological fluid universally known as the optimal post-natal source of nutrition for infants [1,2,3]. It consists not only of essential nutrients (vitamins, minerals, protein), cells, hormones, immunological and immunomodulatory factors (cytokines, immunoglobulin A, microRNAs), but also of non-nutritive bio-molecules (glyco-conjugates, oligosaccharides) [4] and a vast array of microbes (the bacteria, archaea, viruses, protozoa and anaerobic fungi) known as the human milk microbiota [5,6,7].

## 2. Human Breast Milk Bacteriome 

The presence of bacteria in HBM as both an evolutionary strategy subsequent to the divergence of mammals millions of years ago and an effect of natural selection has made it uniquely suited to nourish infants [8]. For example, an infant consuming an average of 800 mL HBM per day has been reported to ingest 10^4^–10^6^ commensal bacteria [9]. These bacteria are not merely present or transient but are rather transcriptionally active and functioning participants in the infant’s gut community [10]. These bacteria serve as a physiological and continuous source of commensal and potential probiotic bacteria to the infant’s gut [2,11,12]. In addition to the role of HBM bacteria in infants, these bacteria also help in maintaining the mother’s health, i.e., aiding in the prevention of mastitis [13].

## 3. Bacterial Diversity in Human Breast Milk

In the pre-2000 era, HBM was considered sterile and devoid of micro-organisms [2]. However, in 2003, Martin et al. described the presence of commensal and probiotic bacteria in HBM. The study used culture-dependent techniques and found, in all samples, a predominance of the lactic acid bacteria *Lactobacillus gasseri* and *Lactobacillus fermentum* [14]. Lactic acid bacteria, including species of genera *Lactobacillus* and *Bifidobacterium*, are of interest in matters of human health. They are known to limit the growth of potential pathogenic organisms in the gastrointestinal tract due to their ability to produce acetate and lactate from the metabolism of ingested sugars. *Bifidobacterium* was not initially regarded as a typical lactic acid bacteria due to their unrelated genetic structure, however, their habitat overlaps with that of lactic acid bacteria and they produce lactic acid as an end-product of fermentation [15]. According to FAO/WHO, select lactic acid bacteria strains with proven probiotic properties are thereby referred to as probiotics [16].

These early descriptions of bacterial diversity in HBM came from utilizing culture-dependent techniques which allowed for the detection of facultative anaerobic bacteria, their close-relatives Gram-positive bacteria and lactic acid bacteria in aseptically collected HBM [9,14]. The more fastidious organisms, such as strict anaerobes which require a more exacting culture media and stricter growth requirements, were not detected [11]. More recently, and only using culture-independent DNA-based techniques including denaturing gradient gel electrophoresis (DGGE), temperature gradient gel electrophoresis (TGGE) and next generation sequencing (NGS), additional bacterial genera have been detected. These include the obligate anaerobes, particularly *Bifidobacterium* spp., *Bacteroides* spp., and members of the Clostridia class [5,17]. 

In the first NGS study of HBM samples, the diversity of bacterial communities, or bacteriomes, was characterized using 454 pyrosequencing to target the 16S rRNA gene. The most abundant genera were found to be *Streptococcus*, *Staphylococcus*, *Serratia* and *Corynebacterium* [5]. In another study by Jost et al. (2013), NGS revealed gut anaerobes including Clostridia whose members produce the metabolite butyrate which helps maintain colon health [11]. The bacterial diversity of HBM over the course of lactation (colostrum, transitional and mature milk) was also characterized using same technique [18]. Several micro-organisms including *Streptococcus* spp., *Staphylococcus* spp. and lactic acid bacteria (*Weisella* spp. and *Leuconostoc* spp.) were found throughout; mature milk samples, however, possessed additional bacterial genera that typically dwell in the oral cavity [18]. Similar bacterial diversity patterns were seen using NGS with Illumina MiSeq [19], as well as in two studies using metagenomic approaches [6,13]. While 16S amplicon approaches target bacteria, metagenomic studies allow for the detection of other microorganisms such as fungi, protozoa, archaea and viruses. Another advantage of metagenomic methods is that they allow for taxonomic identification at the species level, whereas 16S can only confidently identify organisms until the genus level. Firmicutes and Proteobacteria were the dominant phyla observed in both the metagenomic studies and the 16S sequencing studies. At the genus level, however, the relative abundance of *Streptococcus* and *Staphylococcus* was relatively low [6,13]. A systematic review of the HBM bacteriome using culture-independent techniques has revealed that these two genera (*Staphylococcus* and *Streptococcus*) may be universally predominant regardless of differences in geographical area or methodological approach [20].

### 3.1. The Core Milk Bacteriome?

Hunt and co-workers (2011) suggested that there is a “core” HBM bacteriome of nine bacterial genera including *Staphylococcus*, *Streptococcus*, *Serratia*, *Pseudomonas*, *Corynebacterium*, *Ralstonia*, *Propionibacterium*, *Sphingomonas*, and *Bradyrhizobium*. The operational taxonomic units (OTU) were found to represent about half of the observed microbial community, though their relative abundances varied quite significantly among women [5]. Since then, various studies have confirmed the hypothesis of a core bacteriome [13,19,21]. This “core” was not observed across colostrum samples, suggesting that the acquisition of a stable microbial profile is gradual [22]. 

The core bacteriome may consist of species needed for maintaining efficient ecosystem homeostasis whose loss (or gain) may negatively impact the structure and function of other members in the ecosystem [23]. Interestingly, however, it is assumed that the core bacteria are less affected by the environmental factors (diet, obesity, stress) which are known to alter the composition of the other bacteriome [24]. 

**Figure 1:**
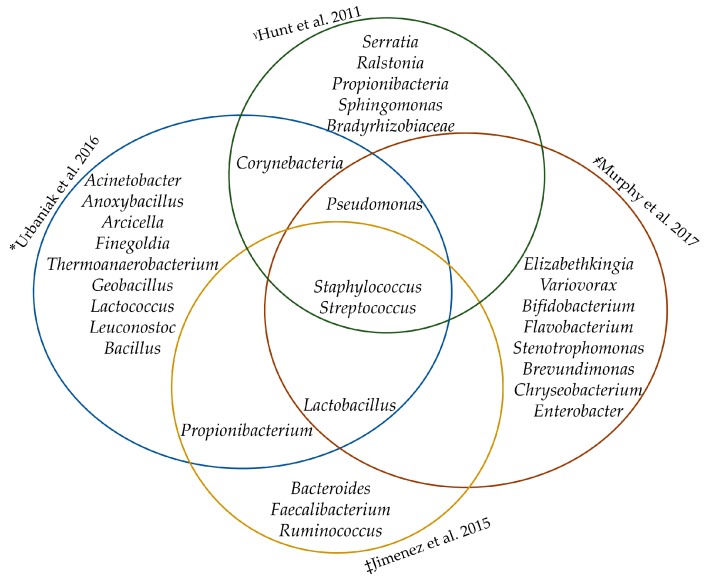
The core human breast milk bacteriome. *QIAamp^®^ DNA Stool Kit (Qiagen), V6 region of bacterial 16S rRNA gene, Ion Torrent platform. ˠQIAmp DNA Stool Mini Kit (Qiagen), V3–V4 region of bacterial 16S rRNA gene, Illumina MiSeq platform. ‡QIAamp DNA Mini Kit (Qiagen) with previous mechanical and enzymatic lysis, V1–V2 region, Pyrosequencing.

Differences in this “core” bacteriome have been reported across various studies [5,19,21]. These differences could be a result of the following factors: sample collection methods (electric pump vs. manual expression, skin cleaning vs. decontamination), use of different DNA extraction kits, storage conditions and freeze/thaw cycles of samples, sequencing platforms, possible biases introduced by the use of primers with the amplification of different 16S rRNA gene hypervariable regions and use of different pipelines in analyzing sequence reads [6,21]. Despite these factors, the identified core bacteria genera commonly included *Staphylococcus*, *Streptococcus*, *Lactobacillus and Propionibacterium* (see Figure 1). Formal meta-analysis of studies characterizing the core HBM bacteriome in different geographical locations, however, is required.

### 3.2. Origin of The Human Breast Milk Bacteriome

There have been several debates about the origin of bacterial communities in HBM. In 2003, Martin et al. used randomly amplified polymorphic DNA (RAPD) polymerase chain reaction (PCR) to analyze lactic acid bacteria from HBM, breast skin and areola. It was observed that the lactic acid bacteria isolated from HBM had DNA profiles that were different from those isolated from either the breast skin or the mammary areola [14]. Obligate anaerobes (*Bacteroides* spp. and/or *Bifidobacterium* spp.) which are unlikely to survive the aerobic conditions of the breast skin have also been isolated from HBM [12,21]. 

In addition, an experiment conducted by Hunt et al. (2011) showed that although the bacteriome in the sebaceous skin and HBM share many of the same phylotypes, differences were found. *Streptococcus*, one of the most abundant genera in HBM samples globally, was only a minor component of the sebaceous skin bacteriome. *Propionibacterium*, on the other hand, reported as one of the most abundant in sebaceous skin genera, was not among the most abundant genera found in HBM samples [5]. Bacteriome found in HBM using Illumina MiSeq were distinct from the areolar skin in both composition and diversity [25]. 

#### 3.2.1. Retrograde Flow

It is possible that some bacteria found in HBM come from the transfer of oral and skin bacteria which enter the mammary ducts during suckling in a process called retrograde flow [26]. This hypothesis was investigated by Ramsay and colleagues who used ultrasound imaging to demonstrate that there is a high degree of retrograde flow of milk from the infants’ mouth back into the mammary ducts during breastfeeding, providing an ideal route for the exchange of bacteria back into the mammary ducts [26]. *Streptococcus*, one of the most abundant bacterial genera in the HBM bacteriome, also dominates the salivary bacteriome [5,27] lending support to the retrograde flow mechanism, however, investigation into whether both bacterial communities share identical species and strains of *Streptococcus* spp. is warranted. 

#### 3.2.2. Gut–Mammary-Axis

Another more recent hypothesis on the origin of the HBM bacteriome is the entero-mammary pathway where non-pathogenic, intestinally derived bacteria may be transported to other locations such as mucosal surfaces of the lactating mammary gland through the endogenous cellular pathway known as the mononuclear cells [2,3,28]. 

The translocation of the gut bacteria to the mammary glands is aided by the physiological and hormonal changes during late pregnancy and the increased permeability of the intestinal epithelial lining [27]. In support of this hypothesis, animal studies have shown increased bacterial translocation of both aerobic and anaerobic organisms from the gut to the mesenteric lymph nodes and mammary glands in pregnant and lactating mice [28]. 

In addition, Zhou et al. have found similar bacterial signatures in the dendritic cells (DC), breast milk (BM), intestines and lymph nodes of lactating mice [29] suggesting translocation of bacteria from the intestines by the DC, into the lymphatic system and carried through to the mammary gland environment.

#### 3.2.3. Mammary Gland Bacteriome

The human breast tissue bacteriome has recently been determined [30,31] from breast tissue biopsies collected from different sites within the breast. The viability of the bacteria was confirmed by culture. As in HBM bacteriome, the principal phylum, Proteobacteria, was the major phylum detected in human breast tissue bacteriome. Moreover, the two microbial communities share several bacteria genera [6,21].

The breast ductal bacteriome has recently been described by analyzing nipple aspirate fluid (NAF) [32]. NAF is regularly secreted by the epithelial cells lining the breast ducts that can be collected non-invasively from the duct in most women by applying negative pressure with a syringe attached to a suction cup [33]. The duct is in constant communication with the external environment through the areola. There is likely to be interaction of microbes between these various compartments in the mammary environment.

Together, we may view the ecological niches in the human bacteriome, not as isolated environments, but as a network of inter-related communities experiencing constant exchange [5]. It seems likely that the HBM bacteriome may be constantly influenced by exposure to other microbial populations associated with mother and child. 

## 4. Factors Which May Affect the Human Breast Milk Bacteriome

Many factors have been identified to contribute to the variability of the HBM bacterial community between different women and within the same woman while she is experiencing different physiological, hormonal and pathological conditions. Both maternal and infant factors have been shown to contribute to this variation. While factors such as infant gender have been shown to have no influence [19], studies have shown that maternal health and geographical location play a major role (see Table 1).

1. Mode of delivery: An estimate by qPCR [34,35] claims that women who delivered via caesarean section (CS) have been shown to have a lower abundance of *Lactobacillus* spp. (*L. fermentum* and *L. salivarius*), *Bifidobacterium* spp. when compared with the higher bacterial counts of women who delivered vaginally. The HBM of mothers who had elective CS also showed decreased members of the family Leuconostocaceae and increased Carnobacteriaceae, when compared with women who delivered vaginally [18]. However, in a study by Urbaniak and colleagues which utilized a more robust statistical analysis [19], no difference in bacterial profiles was observed between women who delivered vaginally and those who delivered via emergency CS. It was suggested that this could be due to the initiation of the labor process, including physiological stress and hormonal signals which may influence increased permeability of intestinal epithelial lining for translocation [18,19].
2. Maternal weight: Higher levels of *Staphylococcus* spp. and lower levels of *Bifidobacterium* spp. were observed in HBM from overweight mothers as compared with normal-weight mothers [36]; and a less diverse bacterial community has also been observed in obese mothers [18]. This may be due to the metabolic capacity of the bacteriome of obese individuals which has an increased capacity to harvest energy from diet [37].
3. Antibiotics and Chemotherapy: A lower abundance of lactobacilli and bifidobacteria was detected in HBM of mothers who were exposed to antibiotics during the perinatal stage [35]. Exposure to anti-cancer chemotherapy also resulted in a reduction of the genera *Bifidobacterium*, *Eubacterium*, *Staphylococcus and Cloacibacterium* [38].
4. Maternal health: Gronlund et al. (2007) described that the bacteriome is influenced by maternal health. In his study using direct PCR analysis, allergic women exhibited a significantly lower *Bifidobacterium* spp. in their BM, with their infants also having lower fecal bifidobacteria counts [39]. African women with HIV–RNA in their HBM had an increased bacterial diversity and higher abundance of *Lactobacillus* spp. compared to controls [40]. Lower abundance of *Bifidobacterium* spp. and *Bacteroides fragilis* group have been detected in HBM of women with celiac disease [41].
5. Lactation stage: A higher bacterial diversity but lower total bacterial count and less bifidobacteria species were detected in colostrum when compared with mature HBM [18]. *Bifidobacterium* spp. and *Enterococcus* spp. counts, along with total bacteria increased as the lactation stage progressed [34]. In a similar study, however, transition milk samples were observed to possess higher diversity than colostrum and mature milk [34,42].
6. Geographical location: The bacterial genera found in HBM of Spanish mothers were different to those of Americans [5,42], or Finnish women [18] using sequencing techniques with a similarly high throughput. In a study by Kumar et al., Chinese women had high levels of Actinobacteria in comparison to the similarly high levels of Bacteroidetes detected in Spanish women [43].
7. Gestational age: *Bifidobacterium* spp. were observed to be higher in HBM of women with term babies than in preterm gestation.

## 5. Plausible Functions of Human Breast Milk Bacteria

The HBM bacterial communities play a role in reduction of the incidence and severity of infections in the breastfed infant via mechanisms such as competitive exclusion and production of antimicrobial compounds. HBM bacteriome also improve the intestinal barrier function by increasing mucin production and reducing intestinal permeability [2,3]. 

### 5.1. Vertical Transmission and Seeding of Infant Gut by HBM Bacteria

Evidence for vertical transmission of maternal bacteria, via milk, to the infant’s gut has been shown in humans [12,14]. *Lactobacillus* spp. sequences isolated from infant feces showed identical patterns to those found in their respective maternal HBM but differed from the profiles found in the maternal vagina [14]. Identical bacterial strains of bifidobacterium (*Bifidobacterium breve* and *Bifidobacterium longum* subsp. longum), and *Lactobacillus plantarum* have been confirmed in HBM and infant feces of mother-infant pairs, suggesting vertical transfer from the mother’s milk to the infant [21,44,45]. 

Recently, additional supporting evidence for vertical transmission of maternal microbes has been published. Shotgun metagenomics was used to demonstrate the transfer of specific strains of *Bifidobacterium* spp., *Ruminococcus bromii*, and *Coprococcus comes* within different mother-infant pairs [10]. Another study compared the fecal bacteriome of breastfed infants, whose gut is dominated by *Bifidobacterium* spp. and *Lactobacillus* spp. transmitted from HBM, to that of formula-fed infants, whose gut is predominantly colonized by enterococci, enterobacteria and *Clostridium difficile*—a pathogen associated with enteric and atopic diseases [46,47].

### 5.2. Anti-Infective Activities of HBM Bacteria

In vitro studies show that *Lactobacillus rhamnosus* and *Lactobacillus crispatus*, isolated from HBM have anti-infective properties against *Staphylococcus aureus (S. aureus). S. aureus* has been implicated in mastitis [9], antibiotic-resistant nosocomial infections, and neonatal infections. HBM-derived lactobacilli strains, particularly *Lactobacillus salivarius (L. salivarius)* CECT5713, produce not only in vitro antibacterial activity, but also a protective effect against *Salmonella enterica* serovar Cholerasuis *(S. enterica)* CECT4155 in animal models. This is mediated through inhibiting the adhesion of *S. enterica* to mucins and increasing the likelihood of survival of infected mice [48]. 

Additionally, HBM lactic acid bacteria protect the physiological environment of the gut through mechanisms such as the production of organic acids and the lowering of pH to inhibit the growth of various facultative and anaerobic bacteria [48,49].

### 5.3. Immuno-Modulatory Activities of HBM Bacteria

HBM bacteria provide a source of bifidobacteria to the infants’ gut. Bifidobacteria in turn activate T-regulatory cells which can result in improved resistance to pathogenic microorganisms [2,46]. In addition, the human milk metagenome has been shown to contain immunomodulatory DNA motifs which may help decrease exaggerated inflammatory responses to colonizing bacteria [6]. HBM derived probiotic strains, *Lactobacillus fermentum* CECT5716 and *Lactobacillus salivarius* CECT5713, have demonstrated in vitro immunomodulatory activity by modulating the activation of peripheral blood mononuclear cell (PBMC) subsets, CD8+ natural killer cells, Treg cells, and several cytokines and chemokines. This effect was not seen with probiotic bacteria of non-milk origin [50].

### 5.4. Anti-Allergic Properties of HBM Bacteria

A beneficial association has been proposed between HBM lactic acid bacteria and allergies. For example, animal studies have shown that probiotic lactobacilli (of HBM origin), *Lactobacillus gasseri* together with *Lactobacillus coryniformis*, decrease the occurrence and severity of allergic responses to cow milk protein [51]. Although a randomized, controlled trial showed that probiotic supplementation in the first six months of life did not reduce the risk of atopic eczema [52], other studies found that supplementation with specific *Lactobacillus* spp. and/or *Bifidobacterium* spp. in mothers led to a reduced infant eczema at one year and two years of age [53,54]. The Hygiene Hypothesis suggests that the anti-allergy properties of probiotics are due to the down-regulation in the production of Th2 cytokines by inducing a Th1 response [51]. Disturbance in the regulation of the immune system is considered an underlying cause of allergies [55].

It is also interesting to note that the presence of viridans streptococci, one of the dominant bacterial groups in HBM, seems to be a feature of a healthy infant gut in contrast to that of atopic infants whose gut is dominated by *Klebsiella* spp. [56]. Animal studies have shown that defective maturation of the immune system in animals that lack appropriate host–microbe interactions during early life makes them prone to allergic immune responses. This defective immune maturation occurs with the introduction of even a single strain of bacteria such as *Bifidobacterium* spp. during the neonatal phase [57]. 

### 5.5. Metabolic Activities of HBM Bacteria

HBM bacteria are essential for the digestion of oligosaccharides (the fourth main component of HBM). Infants lack the necessary enzymes to digest these and, instead, the HBM microbes ingested during feedings break them down into short chain fatty acids (SCFA); this end-product also serves as one of the main energy sources for colonocytes [58]. This is helpful for increased nutrient absorption as the gut of an infant is much shorter than in adults leading to quick transit of food [42].

### 5.6. Anti-Tumor Properties of HBM Bacteria 

HBM bacteriome may have an anti-tumor role. In vitro studies have demonstrated that the heat-killed cells and cytoplasmic fractions of *Enterococcus faecalis* and *Staphylococcus hominis* isolated from HBM possess anti-tumor activity against a breast cancer cell line [59]. Another in vitro study has demonstrated the therapeutic, anti-cancer activity of lactic acid bacteria, *Lactococccus lactis* subsp. *lactis*, against colon cancer [60], This is relevant to both mother and infant health.

## 6. Implications of the Human Breast Milk Bacteriome in Disease

### 6.1. Mastitis

Lactational mastitis is an inflammatory condition of the mammary gland that occurs in about 30% of breastfeeding women [61,62]. It is characterized by local symptoms (localized warmness and soreness on the affected breast, pain, redness, swelling of the breast), in addition to systemic symptoms (fever). It is also a major reason breastfeeding (BF) is discontinued [63,64].

Mastitis is a dysbiosis of the HBM bacteriome characterized by rapid growth of opportunistic pathogenic bacteria including members of *Staphylococcus* and/or *Streptococcus*, Corynebacterium and depletion of commensal bacteria (*Lactococcus* and *Lactobacillus*) [61,65,66]. In acute mastitis, the bacteria count of *Staphylococcus aureus* (*S. aureus*) is said to increase greatly to about 4.0 log10 cfu/mL from 1.5–3.0 log10 cfu/mL in healthy subjects. Another species, *Staphylococcus epidermidis* (*S. epidermidis*), normally appearing on skin and mucosa, is an under-recognized causative agent of lactational mastitis. This lack of recognition may occur if the clinician believes its presence stems from contamination of HBM samples with skin flora [61], however, recent studies have shown it plays an increasing role [13]. 

Coagulase-negative staphylococci (CNS) and *S. epidermidis* have been isolated in HBM of women with chronic mastitis. The chronic nature is because *S. epidermidis* forms biofilms and is resistant to many antibiotics [64]. Recent studies also show that the HBM metagenome in women with mastitis is different based on the stage/type of infection. While *S. aureus* was the most common etiological agent of acute mastitis, *S. epidermidis* was found to be the most common bacteria in subacute mastitis [13]. 

A reduced bacterial diversity and species richness has been observed in HBM of women with mastitis [13,66]. Phage related sequences were also observed in HBM of women with acute mastitis. This is because the virulence factors of *S. aureus* are encoded inside phages, making it easier for the bacteria to evade the host’s immune system [13].

Patel et al. (2017) observed members of the genus *Staphylococcus* to be differentially abundant in sub-acute mastitis. Genes related to bacterial chemotaxis and invasion of epithelial cells, bacterial motility proteins and secretion system were enriched in women having sub-acute and acute mastitis [66]. 

A randomized clinical trial has shown that HBM derived probiotics, *Lactobacillus salivarius* CECT5713 and *Lactobacillus gasseri* CECT5714, when compared with antibiotics [65], are a complementary and effective substitute for the treatment and control of mastitis.

### 6.2. Human Immunodeficiency Virus (HIV)

HBM of women infected with HIV has been shown to have a different bacterial composition compared with non-infected women. An increase in both bacterial diversity and *Lactobacillus* spp. frequency has been observed. In contrast, *Staphylococcus hominis* (*S. hominis*) and *S. aureus* were significantly reduced [40] in HIV-infected women. The reduction of these two *Staphylococcus* species may be due to the inhibitory features of lactic acid bacteria against *S. aureus* [40]. In addition, a pathological or disturbed HIV-positive immune system could be a reason for the observed results. In a similar study, however, there was no difference either in bacterial diversity or in the bacterial profiles between HIV-positive and HIV-negative women [67]. The discrepancies in these results are unknown but could, however, be due to a difference in geological location, methodological differences or the small sample sizes used in the later study.

### 6.3. Cancer

Although there has been no direct study on breast milk bacteriome and (breast) cancer, as women with cancer seldom breastfeed, the microbial ecology of other compartments within the mammary environment such as the breast tissue bacteriome and breast ductal bacteriome (using nipple aspirate fluid) have been linked to cancer [32,68]. 

Microbial dysbiosis was implicated in breast cancer in a study by Urbaniak and colleagues. There was a depletion of the lactic acid bacteria, *Lactococcus* spp. and *Streptococcus* spp., and an increased abundance of *Bacillus* spp., *Staphylococcus* spp. and the family Comamonadaceae and Enterobacteria. *Escherichia coli* isolated from breast cancer patients in the same study induced DNA double-stranded breaks [68].

Bacteria may have a systemic influence in the host, promoting, regulating and training the immune system [69]. Bacteria maintain the health of breast tissue by stimulating resident immune cells. In dysbiosis, a condition characterized by the reduction of specific bacterial taxa, there may be lower immune cell stimulation by such bacteria. This in turn creates an environment which may be conducive for breast tumor formation. Xuan et al. found that compared with the adjacent normal tissue, there was a significant reduction of *Sphingomonas yanoikuyae* in breast cancer patients [70]. In a similar study, a member of the *Sphingomonadaceae* family was enriched in nipple aspirate fluid from healthy controls while *Alistipes* was enriched in breast cancer patients [32]. 

The mechanisms, e.g., inflammation and DNA damage, through which bacteria play a role in cancer should be studied further, using animal models, as it’s unsure whether the association of specific bacteria with tumors could be due to their ability to thrive well in particular milieus or because they cause cancer [71].

## 7. Conclusions

In conclusion, the HBM bacteriome has been shown to play a role in anti-infection, immunomodulation and metabolic activity and is known to be influenced by maternal and socio-economic factors. A better understanding of the factors influencing HBM bacteriome may make it possible to manipulate bacterial communities to improve the health and development outcomes of infants. Lastly, the HBM bacteriome may be associated with, or cause, specific disease conditions.

### Gaps Identified

1. There are very limited animal studies demonstrating the specific role of breast milk bacteria in disease conditions.
2. There are few integrative studies exploring the interplay between HBM bacteriome and its metabolites, and their collective role in diseased condition.
3. There is insufficient data on some of the identified HBM bacterial groups. It isn’t enough to simply characterize the bacterial diversity of HBM; rather, the role that each of the bacterium play in maintaining the microbial ecology of the HBM bacteriome and their role in infant and maternal health must be understood.
4. There are few studies on HBM bacteriome and the factors influencing low socio-economic regions, such as many African settings. These regions also boast less research of infant nutrition and this research is necessary to know if there are any underlying genetic mechanisms involved.

## Figures and Tables

**Table 1:** Factors influencing human breast milk bacteriome.

Factors Influencing Human Breast Milk Bacteriome	Bacteriome	References
***Mode of delivery***		
Caesarean section	↓ *Bifidobacterium* spp., ↑ Proteobacteria, ↓ *Lactobacillus* spp. (*L. fermentum* and *L. salivarius*), ↓ Leuconostocaceae, ↑ Carnobacteriaceae	[18,34,35]
Vaginal delivery	↑ *Bifidobacterium* spp., ↑ *Lactobacillus* spp.	[34,35]
***Maternal weight***		
Overweight mothers	↑ *Staphylococcus*, ↓ *Bifidobacterium*	[36]
Obese mothers	Less diverse bacterial community	[18]
***Antibiotic* and *Chemotherapy***		
Perinatal antibiotics usage	↓ *Lactobacillus/Bifidobacterium*	[35]
Chemotherapy	↓ *Bifidobacterium,* ↓ *Eubacterium,* ↓ *Staphylococcus* and ↓ *Cloacibacterium*	[38]
***Maternal health***		
Allergy	↓ *Bifidobacterium*	[39]
Celiac disease	↓ *Bifidobacterium* spp., ↓ *Bacteroides fragilis* group	[41]
HIV	↑ Bacterial diversity, ↑ *Lactobacillus* spp.	[40]
***Geographical location***		
Spanish women	↑ Bacteroidetes	[43]
Chinese women	↑ Actinobacteria	[43]
***Lactation stage***		
Colostrum	↑ Bacterial diversity, ↓ *Bifidobacterium* spp., ↓ Total bacterial count	[18]
Transition milk	↑ *Bifidobacterium*, ↑ *Enterococcus* spp. counts, ↑ Total bacteria count	[34,42]

HIV: human immunodeficiency virus.
